# Performance appraisal method for rural infrastructure construction based on public satisfaction

**DOI:** 10.1371/journal.pone.0204563

**Published:** 2018-10-04

**Authors:** Chuan Chen, Yibin Ao, Yan Wang, Jiayue Li

**Affiliations:** 1 Business School, Sichuan University, Chengdu, Sichuan, China; 2 College of Environment and Civil Engineering, Chengdu University of Technology, Chengdu, Sichuan, China; 3 Department of Engineering Management, Sichuan College of Architectural Technology, Deyang, Sichuan, China; Universidad de La Frontera, CHILE

## Abstract

Rural infrastructure has rather fixed users compared to urban infrastructure. This study evaluated the effect of rural infrastructure construction from the perspective of farmers. First, this study revised the American Customer Satisfaction Index (ACSI) model and selected a performance appraisal index for rural infrastructure based on this revised model. Then, the study adopted an interpretive structural model (ISM), analyzed the influence of each index factor, and developed a hierarchical directed graph. Finally, based on the mutual-influence relationships among the index factors in the hierarchical directed graph, a performance appraisal analytic network process (ANP) model was established. Based on discussions with rural college students and rural households in Sichuan, China, 246 questionnaires were obtained pertaining to rural infrastructure, and an empirical analysis was conducted. The results indicated that the performance of rural infrastructure construction is not very good. In particular, the full use of infrastructure and its role in improving the environment were found to be the worst. Meanwhile, the possibility of building information transparency and the longitudinal comparison of perceived performance appraisal results were the best. The performance of rural infrastructure construction was evaluated based on the perceptions of the direct users of rural infrastructure, and the relationship between the factors and the weight was measured reasonably. The proposed method was found to be workable and the analysis results reliable and effective.

## Introduction

China’s investment in rural fixed assets has grown significantly over the past 10 years. The rate of growth in 2014 was 2.72 times that of 2005. Growth in rural infrastructure is evident as well: 3,368.3 billion yuan was invested in 2014, which was two times that of 2005 [1]. With increased investment in rural areas, rural construction has produced certain benefits; however, problems with rural infrastructure construction have been highlighted as well. Environmental pollution, for example, is a major problem drawing global attention. As with rapid urban development, will the pursuit of new rural construction come at the expense of the environment? Will rural infrastructure construction meet the needs of agricultural production as well as those of peasants after a certain amount of investment and development? Further investigations are needed to address these questions. As such, there is a need to evaluate the effects of rural infrastructure construction by testing past inputs, which can also provide a theoretical reference for further investment and development in rural areas.

Recent studies on the effects of rural infrastructure have mainly focused on three aspects. The first is the assessment of infrastructure sustainability. Boz et al. developed a framework for evaluating sustainable infrastructure projects, proposed corresponding evaluation criteria, and established an evaluation index system [2]. Domingo assessed the effect of complex medical and health programs on construction-waste generation through interviews with people in the health-care infrastructure as well as questionnaire surveys [3]. Analyzing 23 feasibility study reports, Shen et al. identified 30 key indexes for the sustainable development of infrastructure projects and selected 20 key assessment indexes using fuzzy set theory [4]. Torres-Machi et al. analyzed environmental economic evaluation models as well as the practice of sustainable network pavement management [5]. Reza et al. proposed an evaluation method for sustainable infrastructure development based on the energy value of the whole life cycle [6]. Parrish et al. proposed life-cycle analysis (LCA), which assesses a project’s social, economic, and environmental aspects and requires that all three aspects be balanced to improve sustainability on the basis of life-cycle cost (LCC) [7]. Investigating the infrastructure sustainability of wastewater treatment, Glick et al. evaluated a case using both LCC and LCA, and concluded that most sewage treatment and pollution costs arise from the sewage transport process. They recommended replacing central treatment facility (CTF) technology with community-scale technology (CST) [8]. Analyzing the environmental, economic, and social aspects of four commonly used underground public infrastructure construction methods, Ariaratnam et al. found that pilot tube micro tunneling (PTMT) had the highest sustainability [9]. Bocchini et al. used the comprehensive application of resilience and sustainability to evaluate infrastructure construction [10]. Using system dynamics, Zhou et al. studied the unique characteristics of infrastructure from a microengineering perspective and established a model to analyze sustainable construction and operation [11]. Anagnostopoulos et al. combined geographic information systems (GIS) and spatial fuzzy analytic hierarchy process (SFAHP) to analyze and sort the location of a site [12].

Second, a number of studies have employed social investigation to examine rural infrastructure construction, use of the situation, willingness to raise funds, sources of funds, and user satisfaction. Wang et al. divided rural infrastructure into two types: production and living. They conducted household surveys in 19 villages in Guangdong and analyzed farmers’ satisfaction with the current infrastructure supply and their willingness to finance based on their needs [13]. Through case studies of three infrastructure projects (rural schools, drinking water, and irrigation), Ma et al. analyzed the government’s role in rural infrastructure investment and the direction of self-financing investment among villagers [14]. Yi et al. surveyed 101 villages in 5 provinces to analyze famers’ needs and investment behaviors in relation to roads, irrigation facilities, and drinking water facilities [15]. To analyze the status of private capital involved in rural infrastructure and farmers’ willingness to invest, Gan et al. investigated 31 villages and towns in the resource-rich areas of Shanxi, Shaanxi, and Inner Mongolia [16]. Using data from 30 provinces, Hao et al. analyzed the effect of increased income on rural infrastructure [17]. Using 670 questionnaires, Fan et al. established a structural equation model of the factors that influence satisfaction with rural infrastructure construction [18]. Using Henan Province to study farmers’ willingness to invest in rural infrastructure, Zhang et al. made recommendations for bottom-up and top-down public decision-making mechanisms [19].

Third, some studies have focused on project performance appraisal. Project performance measurement has been widely applied as an important tool for improving the level of enterprise project management. Although appraising the performance of infrastructure construction can improve the efficiency of public management and decision-making, research in this area is limited. To identify the gap between theory and practice, Bassioni et al. reviewed performance appraisal frameworks and their application among construction companies in the United Kingdom [20]. Ramani et al. used performance management to establish a sustainable improvement framework model for traffic management [21]. Conducting questionnaire surveys on the importance of performance indexes for participants in a project in Hong Kong, Lai and Lam found significant differences in the attitudes of participants [22]. Existing studies have mainly used the key performance index method to establish a project performance index system. Bassioni et al. argued that the key performance index method pays too much attention to project performance rather than enterprise performance [20]. Discussing project performance measurement from the perspective of contractors, Zhang et al. noted that research on project performance measurement has mainly focused on the perspectives of third parties and owners [23].

Compared to general construction projects and urban infrastructure construction projects, rural infrastructure construction has clear and fixed end users. Thus, the main target of infrastructure construction should be meeting users’ needs. Accordingly, this study used a customer satisfaction index (CSI) model to evaluate the performance of rural infrastructure construction. Factors that affect the performance of rural infrastructure can be preliminarily determined on the basis of CSI. However, since the relationships between various factors are different from general merchandise, the relationships among various factors in the CSI model cannot be fully applied. In this study, the internal relations between factors were determined using the interpretive structural model (ISM) theory. Project performance index weight is usually determined using the analytic hierarchy process (AHP) method, which reduces problems to a top-down hierarchical structure and assumes that elements on the same level are independent (i.e., no mutual-influence relationship exists). Since factors are not completely independent but mutually influenced, this study employed an analytic network process (ANP) and used Super Decisions (v. 2.8.0) to determine the weight of the indexes in the performance appraisal of rural infrastructure. The purpose of using CSI is to obtain users’ attitudes toward infrastructure. Data were obtained by investigating college students from rural areas in Sichuan Province and then conducting a survey of selected villages. Following analysis and calculation, rural infrastructure construction was evaluated from farmers’ perspectives. On that basis, reasonable, scientific suggestions are proposed for the future planning and construction of rural infrastructure.

The remainder of this paper is organized as follows. Section 2 presents the methods used in the study. Section 3 introduces the empirical study. Next, the empirical study results, discussion, and recommendations are presented in section 4. Finally, section 5 concludes the paper by providing the key findings of the study.

## Methods

### Customer Satisfaction Index (CSI) model

Dardozo introduced customer satisfaction into the marketing field in 1965 [24]. Howard and Sheth (1969) suggested that the evaluation of customer satisfaction is restricted to a certain time or occasion after the purchase [25]. Day and Bodur (1977) defined customer satisfaction as a kind of process that is generated by experience and evaluation [26]. After that, Hunt proposed a value and satisfied-relationship model [27]. In 1989, under the guidance of Professor Fornell at the University of Michigan, the Swedish Customer Satisfaction Barometer (SCSB)—the first national CSI model—was developed [28]. On this basis, CSI models were established in Sweden, the United States, and other countries. The most widely used model is the American Customer Satisfaction Index (ACSI), which was developed in 1994 and covers 10 economic sectors and 43 industries (http://www.theacsi.org/about-acsi/history). In 1999, the China Quality Association, Peking University, Tsinghua University, the People’s University, the Academy of Social Sciences, and other institutions jointly designed a CSI evaluation system. As China’s first standardized evaluation method, it laid the theoretical foundation for future studies of public satisfaction. Since then, scholars have thoroughly studied the customer satisfaction evaluation index system and the customer satisfaction model [29,30]. Revising the ACSI model, Li applied it to rural public infrastructure satisfaction and developed an evaluation index system for farmers’ satisfaction with rural public infrastructure [31,32]. In 2013, Ma Jieqiong used the improved ACSI to evaluate the performance of public projects and encourage the public to participate in performance appraisals of local government projects [33].

Based on the literature review, the CSI model includes seven indexes: expected quality, perceived product quality, perceived service quality, perceived value, customer satisfaction, customer complaints, and customer loyalty.

### Interpretive Structural Modeling (ISM)

Interpretive structural modeling (ISM) was developed by Warfield to analyze complex system structures [34]. The core idea of ISM is to extract the constituent elements of problems and use some auxiliary means—such as matrices, programming, and direct graphs—to deal with the relationships between factors. This allows us to obtain a clear hierarchical structure and hierarchical structure graph, as follows:

- Determine the research object and determine the relationships between the various factors of the study object through an ISM analysis team composed of experts in the relevant industry. Compose element set *S* so that element set *S* = {*S*_1_,*S*_2_,…,*S*_*n*_}.
- After determining the factors affecting the relationship, determine the direct relationship between the two elements according to the following:
  $$S_{i}RS_{j}\left\{ \begin{array}{c} =1,S_{i}has\;a\;direct\;dualistic\;relationship\;wiht\;S_{j}\\ =0,S_{i}does\;not\;has\;a\;dualistic\;relationship\;with\;S_{j} \end{array}\right.(i,j=1,2,\ldots n).$$
  The adjacency matrix is established according to the above relation: *A* = (*a*_*ij*_)*n* × *n*.

- The reachable matrix is calculated by the adjacency matrix, and the hierarchical structure of the index factor is drawn.

### Analytic Network Process (ANP)

Analytic network process (ANP) is a decision-making method proposed by Saaty, which is suitable for a nonindependent hierarchical structure. Although based on the analytic hierarchy process, this method is more flexible [35,36].

Unweighted super matrix *W*

Assuming the control layer of ANP has *P*_1_,*P*_2_,…,*P*_*N*_, and the network layer has an element level *S*_1_,*S*_2_,…,*S*_*N*_, where there are elements *s*_*i*1_,*s*_*i*2_,…,*s*_*iN*_ in *S*_*i*_, in which the control layer element *P*_*i*_(*i* = 1,2,…,*m*) is the criterion, *S*_*jk*_(*k* = 1,2,…,*n*_*j*_) is the substandard, and the element in the element set *S*_*i*_ carries the indirect advantage of comparison to draw the matrix according to its influence on *S*_*jk*_. The characteristic root method obtains the sort vector [*w*_*i*1_^(*jk*)^*w*_*i*2_^(*jk*)^…*w*_*in*_^(*jk*)^]^*T*^ according to the consistency check and obtains matrix *w*_*ij*_:

$$W_{ij}=[\begin{array}{ccc} {w_{i1}}^{\left(j1\right)} & {w_{i2}}^{\left(j2\right)} & \begin{array}{cc} \ldots & {w_{i2}}^{\left(jn_{j}\right)} \end{array}\\ {w_{i2}}^{\left(j1\right)} & {w_{i2}}^{\left(j2\right)} & \begin{array}{cc} \ldots & {w_{i2}}^{\left(jn_{j}\right)} \end{array}\\ \begin{array}{c} \vdots \\ {w_{in_{i}}}^{\left(j1\right)} \end{array} & \begin{array}{c} \vdots \\ {w_{in_{i}}}^{\left(j2\right)} \end{array} & \begin{array}{cc} \begin{array}{c} \vdots \\ \ldots \end{array} & \begin{array}{c} \vdots \\ {w_{in_{i}}}^{\left(jn_{j}\right)} \end{array} \end{array} \end{array}].$$ (1)

The column vector *W*_*ij*_ is the ranking vector of the important degree of $S_{i1},S_{i2},\ldots ,S_{in_{i}}$ in *U*_*i*_ to $S_{i1},S_{i2},\ldots ,S_{in_{j}}$ in *S*_*j*_. If *U*_*j*_ is not affected by *S*_*i*_, then *W*_*ij*_ = 0. Using the above steps, the super matrix *W* of *P*_*s*_ can be calculated as follows:

$$W=[\begin{array}{ccc} W_{11} & W_{12} & \begin{array}{cc} \ldots & W_{1N} \end{array}\\ W_{21} & W_{22} & \begin{array}{cc} \ldots & W_{2N} \end{array}\\ \begin{array}{c} \vdots \\ W_{N1} \end{array} & \begin{array}{c} \vdots \\ W_{N2} \end{array} & \begin{array}{cc} \begin{array}{c} \vdots \\ \ldots \end{array} & \begin{array}{c} \vdots \\ W_{NN} \end{array} \end{array} \end{array}].$$ (2)

Weighted super matrix $\bar{W}$

Comparing the importance of any two elements of *P*_*i*_, we get the weighted matrix

$$A=[\begin{array}{ccc} a_{11} & a_{12} & \begin{array}{cc} \ldots & a_{1n} \end{array}\\ a_{21} & a_{22} & \begin{array}{cc} \ldots & a_{2n} \end{array}\\ \begin{array}{c} \vdots \\ a_{n1} \end{array} & \begin{array}{c} \vdots \\ a_{n2} \end{array} & \begin{array}{cc} \begin{array}{c} \vdots \\ \ldots \end{array} & \begin{array}{c} \vdots \\ a_{nn} \end{array} \end{array} \end{array}].$$ (3)

Then, we get the weighted super matrix:

$$\bar{W}=({\bar{W}}_{ij})=a_{ij}w_{ij},i=1,2,\ldots ,N;j=1,2,\ldots ,N.$$ (4)

### Performance appraisal system method

**Fig 1:**
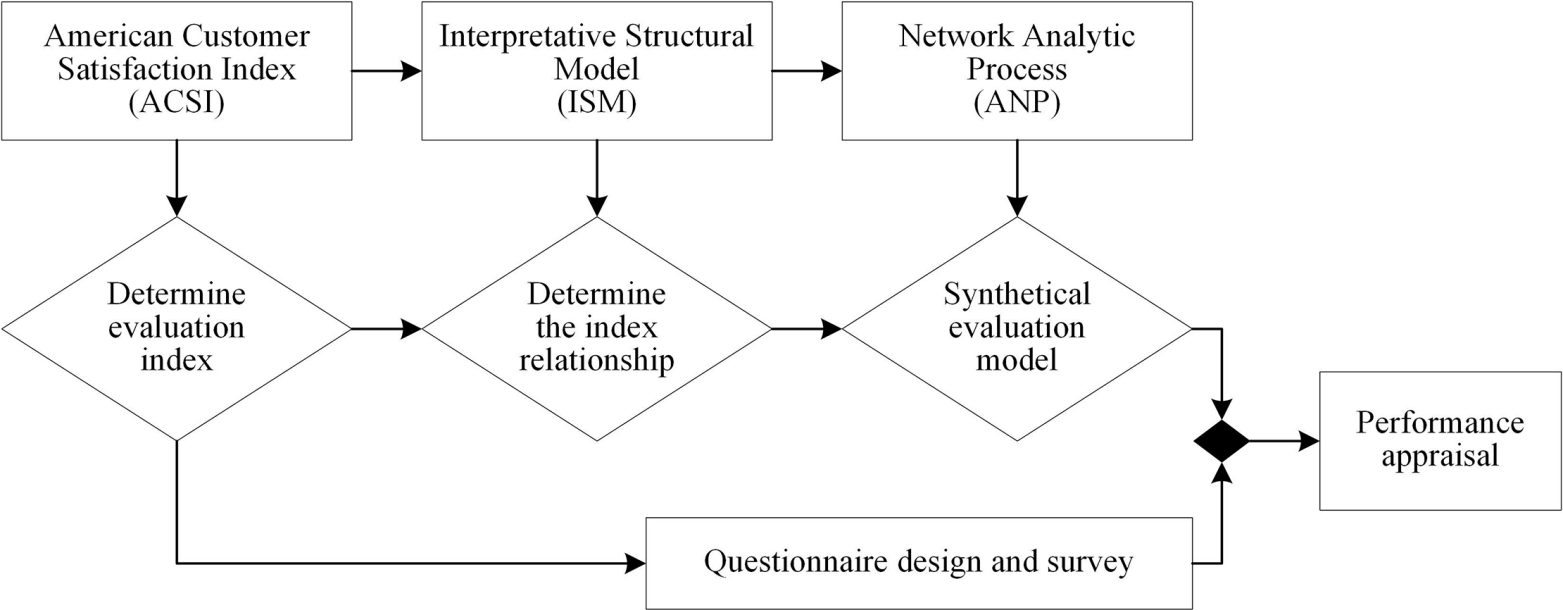
Performance appraisal process.

This study appraised rural infrastructure construction performance based on public satisfaction using an evaluation index system based on ACSI according to research reviews of the multicriteria assessment of sustainable infrastructures [37–39]. Then, the relationship between the indexes was analyzed using structural modeling, and the hierarchical structure of the index factors was obtained. To establish the network hierarchy model and determine the weight of each evaluation index, household survey questionnaires were designed based on performance appraisal indexes, which were selected based on the literature review and expert investigation. Then, the performance of rural infrastructure construction was evaluated based on the household perception data. The performance appraisal process is shown in Fig 1.

## Empirical study

### Revising ACSI to determine the evaluation index

In applying ACSI to the study of rural infrastructure satisfaction, scholars have only used the relevant indexes mentioned in ACSI. There has been no research on the correlation between the indexes. This study suggests that there are internal relations among factors based on the revised ACSI model proposed by Wenyi Li. Although these relationships are not recognized by farmers, they affect farmers’ perceptions of rural infrastructure. Therefore, this study initially assumed that the ACSI indexes for rural infrastructure were interrelated and mutually influenced.

#### Preliminary evaluation index selection

The index design for rural infrastructure construction performance was evaluated based on the revised ACSI model. Twenty-six performance appraisal indexes for rural infrastructure construction were identified by studying related documents. Six new factors affecting famers’ evaluations were obtained through discussions with college students from rural areas; thus, the number of performance appraisal indexes for rural infrastructure construction was 32. The initial selection of the index system is shown in Table 1. Explanations are given below for the six extra indexes proposed in this study (indexes extracted from the literature are not explained).

**Table 1:** The screening results for the evaluation index.

Performance index	Weight	Source	Result	Code
Overall impression of quality	2581	[32]	√	C11
Impression of full use	1896	The author	√	C12
Overall expectation	2366	[32]	√	C21
Expectation of improving quality of life	2124	[32]	√	C22
Expectation of increasing production	478	[32]	×	
Expectation of improving environment	1764	[32]	√	C23
Expectation of increasing income	2198	[32]	√	C24
Total quality perception	2488	[32]	√	C31
Sense of improving quality of life	2068	[32]	√	C32
Sense of increasing production	613	[32]	×	
Sense of improving environment	1476	[32]	√	C33
Sense of improving income	1924	[32]	√	C34
Sense of using security	623	[33]	×	
Sense of reasonable planning and design	517	[33]	×	
Horizontal comparison	1971	[18]	√	C35
Longitudinal comparison	2083	[18]	√	C36
Construction efficiency	602	The author	×	
Sense of quality under given costs	2137	[32]	√	C41
Sense of cost under given quality	2049	[32]	√	C42
Overall satisfaction	2477	[32]	√	C51
Satisfaction relative to expectation	1956	[32]	√	C52
Satisfaction relative to ideal condition	1882	[25]	√	C53
Complaints to others	1328	[34]	√	C61
Frequency of hearing complaints	216	The author	×	
Complaints to relevant departments	2127	[34]	√	C62
Possibility of no longer using rural infrastructure	395	[33]	×	
Possibility of improving the quality of projects	1279	[34]	√	C71
Possibility of participating in construction	572	[34]	×	
Possibility of investment	2234	The author	√	C72
Possibility of participating in operation and maintenance	1849	[34]	√	C73
Possibility of building information transparency	1138	The author	√	C74
Possibility of no corruption	983	The author	√	C75

Impression of full use: Rural infrastructure is the foundation of the development of rural economies. Further, its construction has for a long time been a matter of top-down, government-led supply. Whether rural infrastructure construction meets the real needs of farmers is rarely considered. During the field trip, many farmers suggested that some rural infrastructures are “vanity projects” built for the purpose of local government performance. These are not based on need and are not put into use. Impression of full use is an important part of rural infrastructure performance appraisal and can reflect the construction and use of rural infrastructure.

Construction efficiency: Infrastructure construction has several characteristics (large-scale investment, long construction period, etc.). Further, the project-approval cycle is longer since it involves the use of funds. Some farmers mentioned these problems during the field trip. Construction efficiency directly affects the construction and use of infrastructure.

Frequency of hearing complaints: Li et al. used complaints as an evaluation index. However, this study found that many farmers did not have a deep sense of self-perception regarding this problem, but they clearly perceived the frequency with which other people complained. Thus, this study used the frequency of hearing complaints as a supplementary index.

Investment possibilities: Investment involves risks. Although villagers invest in the construction of some infrastructures for their own use, investment can fail for reasons such as government credit risk, risks in the use of funds, or construction risks. Villagers’ investment possibilities can reflect their confidence in the local government’s ability to deal with risks. The greater the willingness to invest, the greater the confidence in local government.

Possibility of building information transparency: Infrastructure construction involves decision-making, planning, bidding, and many other procedures that pertain to the vital interests of farmers. These include the question of whether to solicit the views of farmers or whether to choose a reasonable contractor. All of these aspects directly affect the results of rural infrastructure construction. Some farmers know little or nothing about rural infrastructure construction. This study used these indexes in the performance evaluation of rural infrastructure construction. The smaller the possibility of the result, the smaller the farmers’ trust in local government.

Possibility of no construction corruption: Construction corruption is a prominent topic in China. Where it exists, it directly affects the quality, efficiency, and quantity of rural infrastructure construction.

#### Evaluation index selection and determination

First, a questionnaire survey was conducted to screen the primary indexes and avoid selecting indexes that pertained to one-sided, repeated, or related issues. Thirty-two preliminary evaluation indexes were selected from the relevant literature and discussions with rural students (see Table 1). Respondents were asked to select 30 indexes in order from the 32 indexes according to the size of the impact on infrastructure performance appraisal. To avoid misleading respondents with the original target sequence, the order of the 32 indexes was randomized for each questionnaire. The weighted cumulative principle was as follows: respondents selected 30 indexes from the 32 indexes, assigned 30 to the first one, 29 to the second, and so on. Finally, all weights were accumulated for each index selected by all 91 respondents according to the order they chose. For example, if all of the 91 respondents selected “impression of full use” as the first one, the weighted cumulative value was 91 * 30 = 2,730. This study excluded weighted cumulative values of less than 900 (a significant fault existed in data below 900) and selected the remaining effective evaluation indexes for the evaluation index system. The screening results for the evaluation index are shown in Table 1.

To ensure respondents were familiar with rural infrastructure construction, the questionnaire was conducted at a local feasibility report review meeting concerning 10 local infrastructure projects. The participants included leaders of the administrative departments for the construction, review experts, representatives of the owners, and representatives of the unit preparing the feasibility report. One hundred questionnaires were issued; 91 were valid (91% effective recovery rate), and the feedback was good. The final composition of the questionnaire is shown in Table 2.

**Table 2:** Final composition of the questionnaire.

NO.	Investigation objects	Number	Ratio
1	Leaders of the administrative departments for construction	13	14.29%
2	Review experts	44	48.35%
3	Representatives of the owners	16	17.58%
4	Representatives of the unit preparing the feasibility report	18	19.78%
5	Total	91	100%

### ISM to determine the index relationship

As shown in Table 1, the final 24 selected indexes were the objects to be studied. Construction administrative departments, experts, owners’ representatives, and representatives of the feasibility report comprised the ISM analysis group. Assuming a relationship existed between the 24 indexes, thus constituting the set of elements *S*, the number of element set *S* = {S_1_, S_2_, …, S_24_} = {C_11_, C_12_, …, C_75_}.

A two-dimensional questionnaire was established according to the ISM principle using the selected 24 indexes. The 91 respondents were asked to judge the relationship between any two indexes. Using statistics, it was confirmed that the two factors had an impact if more than half of the questionnaire showed that the two factors had relationships. The final determination of the ISM adjacency matrix could be obtained from the statistics. The reachable matrix of the adjacency matrix was solved using MATLAB, [36] as shown in Table 3.

**Table 3:** Reachable matrix A.

C	C_11_	C_12_	C_21_	C_22_	C_23_	C_24_	C_31_	C_32_	C_33_	C_34_	C_35_	C_36_	C_41_	C_42_	C_51_	C_52_	C_53_	C_61_	C_62_	C_71_	C_72_	C_73_	C_74_	C_75_
C_11_	0	1	1	1	1	1	1	1	1	1	1	1	1	1	1	1	1	1	1	1	1	1	1	1
C_12_	0	0	1	0	1	0	1	1	1	0	1	1	1	1	1	1	1	1	1	1	1	1	1	1
C_21_	0	0	0	0	1	0	1	1	1	0	1	1	1	1	1	1	1	1	1	1	1	1	1	1
C_22_	0	0	0	0	0	0	0	1	0	0	1	1	1	1	1	1	1	1	1	1	1	1	1	1
C_23_	0	0	0	0	0	0	1	1	1	0	1	1	1	1	1	1	1	1	1	1	1	1	1	1
C_24_	0	0	0	0	0	0	0	0	0	1	1	1	1	1	1	1	1	1	1	1	1	1	1	1
C_31_	0	0	0	0	0	0	0	1	0	0	1	1	1	1	1	1	1	1	1	1	1	1	1	1
C_32_	0	0	0	0	0	0	0	0	0	0	1	1	1	1	1	1	1	1	1	1	1	1	1	1
C_33_	0	0	0	0	0	0	1	1	0	0	1	1	1	1	1	1	1	1	1	1	1	1	1	1
C_34_	0	0	0	0	0	0	0	0	0	0	0	0	1	1	1	1	1	1	1	1	1	1	1	1
C_35_	0	0	0	0	0	0	0	0	0	0	0	1	1	1	1	1	1	1	1	1	1	1	1	1
C_36_	0	0	0	0	0	0	0	0	0	0	0	0	1	1	1	1	1	1	1	1	1	1	1	1
C_41_	0	0	0	0	0	0	0	0	0	0	0	0	0	1	1	1	1	1	1	1	1	1	1	1
C_42_	0	0	0	0	0	0	0	0	0	0	0	0	0	1	0	0	0	1	1	1	1	1	1	1
C_51_	0	0	0	0	0	0	0	0	0	0	0	0	0	0	0	1	1	1	1	1	1	1	1	1
C_52_	0	0	0	0	0	0	0	0	0	0	0	0	0	0	0	0	0	1	1	1	1	1	1	1
C_53_	0	0	0	0	0	0	0	0	0	0	0	0	0	0	0	0	0	1	1	1	1	1	1	1
C_61_	0	0	0	0	0	0	0	0	0	0	0	0	0	0	0	0	0	0	1	1	1	1	1	1
C_62_	0	0	0	0	0	0	0	0	0	0	0	0	0	0	0	0	0	0	1	1	1	1	1	1
C_71_	0	0	0	0	0	0	0	0	0	0	0	0	0	0	0	0	0	0	0	0	0	0	1	1
C_72_	0	0	0	0	0	0	0	0	0	0	0	0	0	0	0	0	0	0	0	0	0	1	1	1
C_73_	0	0	0	0	0	0	0	0	0	0	0	0	0	0	0	0	0	0	0	0	0	0	1	1
C_74_	0	0	0	0	0	0	0	0	0	0	0	0	0	0	0	0	0	0	0	0	0	0	0	0
C_75_	0	0	0	0	0	0	0	0	0	0	0	0	0	0	0	0	0	0	0	0	0	0	1	0

### Establishing ANP model to determine the weight of indexes

**Fig 2:**
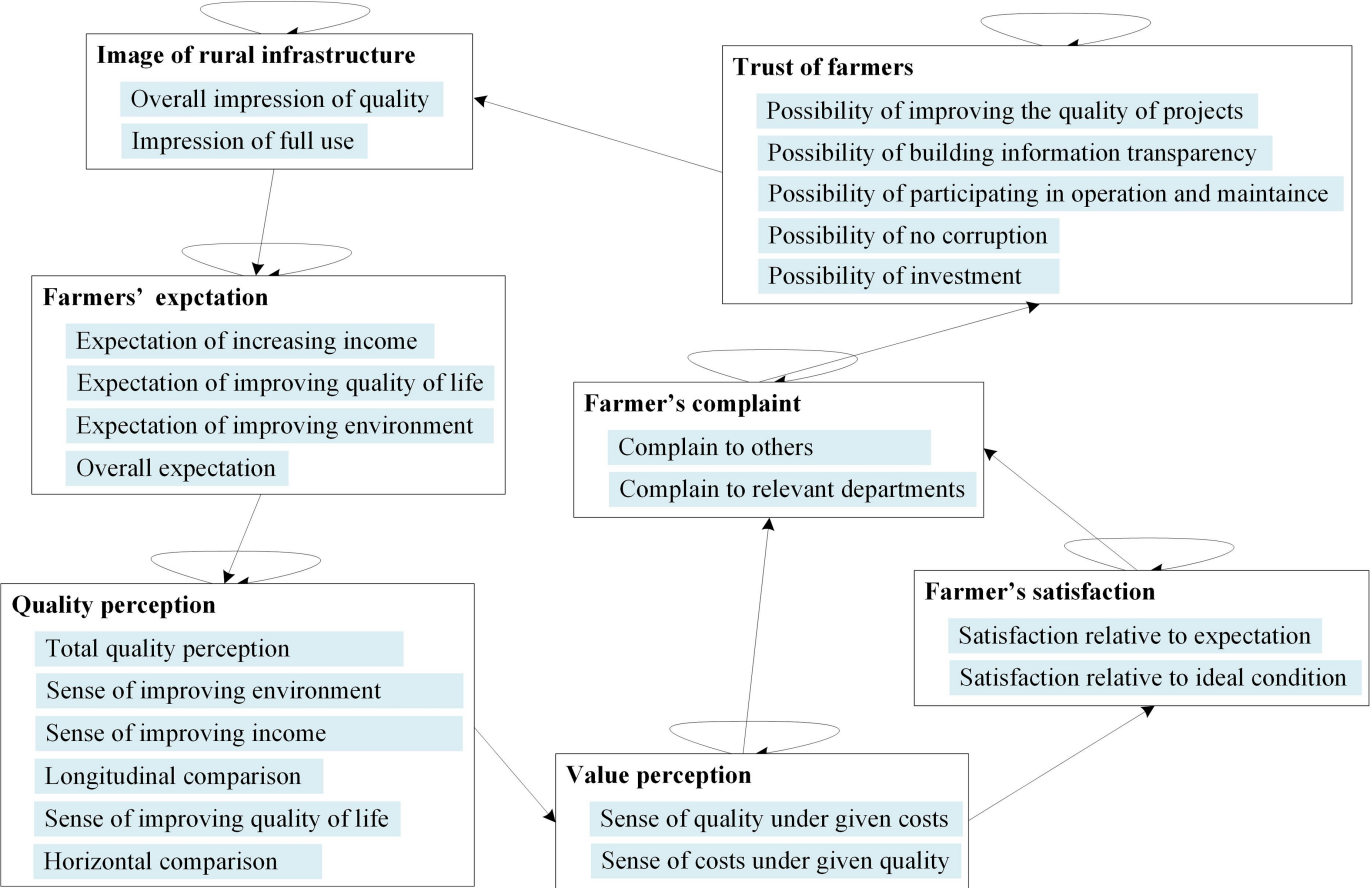
ANP model diagram for rural infrastructure construction performance appraisal.

In the network-level analysis model, the two-level subindex system was the control layer, and the three-level index system was the network layer. The index factors of the network layer were determined according to Table 3. The performance appraisal model for rural infrastructure construction (Fig 2) was obtained by entering the relationships between the indexes into Super Decisions (v. 2.8.0).

The index was established using Super Decisions. First, we needed to compare the indexes of two points. The score value was determined according to the ratio of weighted cumulative values in Table 2, reflecting the impact of 91 expert opinions regarding the construction of rural infrastructure. When the ratio was in the 1.0–1.5 range, the score value was 2; in the 1.5–2.0 range, it was 3; and in the 2.0–2.5 range, it was 4. Each index factor and its related factors needed to be compared according to the weighted cumulative values in Table 2. After each scoring comparison, a consistency test was conducted. After examination, all of the related factors were compared, and the results were satisfactory at CR < 0.1, which met the requirements of the consistency check. Using Super Decisions, we obtained the rural infrastructure performance appraisal for the specific weights of the 24 factors (Table 4).

**Table 4:** Performance appraisal index system factor weights.

Performance appraisal index	Normalized By Cluster	Limiting
**Image of rural infrastructure**		
Overall impression of quality (C11)	0.60000	0.068232
Impression of full use (C_12_)	0.40000	0.045488
**Farmers’ expectations**		
Overall expectation (C_21_)	0.46154	0.045488
Expectation of improving quality of life (C_22_)	0.07692	0.007581
Expectation of improving environment (C_23_)	0.30769	0.030325
Expectation of increasing income (C_24_)	0.15385	0.015163
**Quality perception**		
Total quality perception (C_31_)	0.19187	0.041647
Sense of improving quality of life (C_32_)	0.21437	0.046531
Sense of improving environment (C_33_)	0.03493	0.007581
Sense of improving income (C_34_)	0.06986	0.015163
Horizontal comparison (C_35_)	0.24449	0.053069
Longitudinal comparison (C_36_)	0.24449	0.053069
**Value perception**		
Sense of quality under given costs (C_41_)	0.72125	0.068232
Sense of cost under given quality (C_42_)	0.27875	0.026371
**Farmers’ satisfaction**		
Overall satisfaction (C_51_)	0.49999	0.041861
Satisfaction relative to the expectation (C_52_)	0.33334	0.027908
Satisfaction relative to ideal condition (C_53_)	0.16667	0.013954
**Farmers’ complaints**		
Complaints to others (C_61_)	0.50000	0.068232
Complaints to relevant departments (C_62_)	0.50000	0.068232
**Trust of farmers**		
Possibility of improving the quality of projects (C_71_)	0.06667	0.017058
Possibility of investment (C_72_)	0.20000	0.051174
Possibility to participate in operation and maintenance (C_73_)	0.20000	0.051174
Possibility of building information transparency (C_74_)	0.26667	0.068232
Possibility of no corruption (C_75_)	0.26667	0.068232

### Performance appraisal of rural infrastructure

We used the performance appraisal index system for rural infrastructure to design a questionnaire for farmers. To obtain effective, real data and ensure that respondents understood all of the problems in the questionnaire, the research group organized rural students from engineering management as respondents and visitors to gather the data. A meeting was organized to help students fully understand the questionnaire and to collect data about student attitudes toward rural infrastructure. Then, these students conducted investigations in their hometowns to gather firsthand data. Out of 300 questionnaires issued, 246 were recovered, for a recovery rate of 82%, which is quite high. Among them, 144 respondents were male and 102 were female. The questionnaires covered 23 cities in Sichuan Province and had a wide coverage area.

The reliability of the questionnaire was tested using the Cronbach alpha coefficient (α = 0.8103) with the use of SPSS 21.0, indicating that the questionnaire has high reliability. The questionnaire distribution and recovery numbers are shown in Table 5.

**Table 5:** Questionnaire distribution and recovery.

Region	Number	Region	Number	Region	Number	Region	Number	Region	Number
Bazhong	11	Guangyuan	6	Mianyang	17	Aba State	7	Suining	7
Chengdu	33	Leshan	7	Nanchong	10	Chi Yi Autonomous County	5	Yaan	12
Dazhou	14	Liangshan State	21	Neijiang	7	Ganzi State	7	Ziyang	9
Deyang	9	Luzhou	6	Panzhihua	9	Yibin	15	Zigong	8
Guangan	14	Meishan	7	Mianzhu	5				

Twenty-four questions were set for the 24 indexes for the performance appraisal of rural infrastructure construction, each with five options according to the satisfaction situation, degree of implementation, and possibility of improvement. After preliminary statistics, topics were selected as shown in Table 6.

**Table 6:** Performance appraisal index score table.

Performance appraisal index	Questionnaire score	Model weight	Performance score
**Image of rural infrastructure**			
Overall impression of quality (C11)	4.6446	0.0682	0.3169
Impression of full use (C_12_)	3.3817	0.0455	0.1538
**Farmers’ expectations**			
Overall expectation (C_21_)	4.2397	0.0455	0.1929
Expectation of improving quality of life (C_22_)	4.9835	0.0076	0.0378
Expectation of improving environment (C_23_)	4.9091	0.0303	0.1489
Expectation of increasing income (C_24_)	4.6281	0.0152	0.0702
**Quality perception**			
Total quality perception (C_31_)	4.6364	0.0416	0.1931
Sense of improving quality of life (C_32_)	4.9753	0.0465	0.2315
Sense of increasing production (C_33_)	3.9421	0.0076	0.0299
Sense of improving income (C_34_)	4.4815	0.0152	0.0680
Horizontal comparison (C_35_)	5.0579	0.0531	0.2684
Longitudinal comparison (C_36_)	6.1901	0.0531	0.3285
**Value perception**			
Sense of quality under given costs (C_41_)	4.7190	0.0682	0.3220
Sense of cost under given quality (C_42_)	4.4711	0.0264	0.1179
**Farmers’ satisfaction**			
Overall satisfaction (C_51_)	4.5885	0.0419	0.1921
Satisfaction relative to the expectation (C_52_)	4.4357	0.0279	0.1238
Satisfaction relative to ideal condition (C_53_)	4.1276	0.0140	0.0576
**Farmers’ complaints**			
Complaints to others (C_61_)	4.5270	0.0682	0.3089
Complaints to relevant departments (C_62_)	4.4380	0.0682	0.3028
**Trust of farmers**			
Possibility of improving the quality of projects (C_71_)	5.3568	0.0171	0.0914
Possibility of investment (C_72_)	6.0413	0.0512	0.3092
Possibility to participate in operation and maintenance (C_73_)	5.3058	0.0512	0.2715
Possibility of building information transparency (C_74_)	4.8257	0.0682	0.3293
Possibility of no corruption (C_75_)	4.3112	0.0682	0.2942

According to the questionnaire recycling statistics, measurement was carried out using a Likert-type scale. In Table 7, not satisfied (very small) (much) = 1, less satisfied (smaller) (larger) = 3, general = 5, satisfactory (larger) (smaller) = 7, and very satisfactory (great) (very small) = 9. Each index corresponds to the final questionnaire to obtain its score (*x*_*ij*_ is the number of respondents who selected the score *j* for each index):

$$y_{i}=(x_{i1}\times 1+x_{i2}\times 3+x_{i3}\times 5+x_{i4}\times 7+x_{i5}\times 9)/\sum_{j=1}^{5}x_{ij}.$$

**Table 7:** Questionnaire statistics.

No.	Option Question	Not satisfied (very small) (much)	Less satisfied (smaller) (larger)	General	Satisfactory (larger) (smaller)	Very satisfactory (great) (very small)	Empty	Total
1	What do you think of the overall quality of existing infrastructures?	15	59	125	40	3	4	246
2	Are you satisfied with the village infrastructure’s role?	67	82	75	13	4	5	246
3	What do you expect to be the degree of village infrastructure?	29	61	128	21	3	4	246
4	To what extent do you expect to improve quality of life by improving infrastructure?	9	53	117	57	6	4	246
5	How much do you hope to improve the village’s appearance through the improvement of infrastructure in the rural environment?	11	62	104	57	8	4	246
6	To what extent do you expect to increase household income by improving infrastructure?	23	57	112	42	8	4	246
7	What do you think of the overall quality of rural infrastructure construction?	18	64	110	44	6	4	246
8	How much do you think the construction of rural infrastructure has improved quality of life among villagers?	14	45	118	62	4	3	246
9	What do you think of the effects of infrastructure in terms of improving the village and rural environments?	36	92	83	26	5	4	246
10	How much does infrastructure construction increase farmers’ incomes?	18	70	114	39	2	3	246
11	Are you satisfied with infrastructure construction in your village compared to other villages?	23	39	109	50	21	4	246
12	Are you satisfied with the current infrastructure compared to five years ago?	9	19	71	105	38	4	246
13	Are you satisfied with the quality of rural infrastructure under existing infrastructure costs?	16	64	108	46	8	4	246
14	Are you satisfied with the use of infrastructure under the quality of existing infrastructure?	28	64	103	38	9	4	246
15	Are you satisfied with the overall situation of the village’s infrastructure?	18	71	101	49	4	3	246
16	Are you satisfied with the village’s infrastructure construction compared with your expectations?	24	67	107	39	4	5	246
17	Are you satisfied with the village’s infrastructure construction compared with your ideal condition?	43	59	104	35	2	3	246
18	How much do you complain about village infrastructure?	23	57	120	36	5	5	246
19	How likely are you to complain to related authorities?	33	49	119	35	6	4	246
20	How likely do you think it is that the overall infrastructure condition will be improved?	14	31	107	76	13	5	246
21	Are you willing to invest in rural infrastructure construction?	7	23	82	97	33	4	246
22	Are you willing to help operate and maintain rural infrastructure?	13	25	134	52	18	4	246
23	What do you think about the possibility of village infrastructure construction information becoming more transparent in the future?	24	48	100	63	6	5	246
24	What do you think about the possibility of no corruption in the infrastructure construction in the future?	41	54	99	41	6	5	246

The questionnaire design question was made to correspond to the rural infrastructure performance appraisal index for the weight of ω_*i*_; then, the index considered the weight score of μ_*i*_ = ω_*i*_ × *y*_*i*_. The final scores for the indexes are shown in Table 7.

For the Sichuan area, the performance of rural infrastructure construction, evaluated from the perspective of farmers, is:

$$U=\sum_{i=1}^{24}\mu_{i}=4.7606.$$

## Discussion and suggestions

This study adopted the American Customer Satisfaction Index (ACSI) as the basis for appraising the performance of rural infrastructure. This research method has excellent applicability. The direct users of rural infrastructure are relatively fixed, and they have an intuitive and objective understanding of rural infrastructure; thus, the results of their appraisals are scientific and reasonable. Construction and investment in rural infrastructure aim to promote rural economic development, production, and livelihoods. As such, the construction of rural infrastructure should meet the needs of farmers for both production and daily life. In recent decades, China’s investment in rural infrastructure has increased, and new rural construction has shown initial successes. However, does such construction really meet the most urgent needs in rural areas? Does it really make farmers satisfied or meet the requirements for rural production? Considering these questions, this study evaluated the effect of such construction from the perspective of farmers.

The performance appraisal index for rural construction was based on not only previous studies but also information added through new research. The preliminary determination of the index system is more comprehensive and practical for reflecting farmers’ requirement information for rural infrastructure. To avoid high correlations between indexes, repeated meanings, and poor accuracy measurement, this study administered the index-selection questionnaire to senior professionals. The respondents had high professional levels, including the leaders of construction administration, representatives of rural infrastructure projects, and experts on project evaluation. Thus, the survey results are reliable.

Cumulative weight value can comprehensively reflect how important each index is for performance appraisal. This study used cumulative weight value to select the evaluating index. A pairwise comparison of associated factors was performed using an analytic network process (ANP) based on the AHP method used by Jin et al. to select rural building sites [40]. In addition, this study used index scoring related to cumulative weight value to avoid one-sidedness and single-expert subjectivity. Thus, the index selection and scoring are reliable.

Previous studies have used a customer satisfaction index (CSI) for the performance appraisal of construction projects. However, they only considered seven CSI factors, or just revised the factors, and they did not study the logical relationships between them. This study assumed that farmers’ perception factors are related to each other; thus, it was necessary to study the relationships among them. The relationships among various indexes were studied using an interpretive structural model (ISM). The adjacency matrix of the evaluation index was obtained from the summary statistics by collecting questionnaires on related factors from the abovementioned professionals. MATLAB was used to calculate the reachable matrix; the relationships among the various factors were measured using Super Decisions. Based on participation by professionals, the model is objective, scientific, and reasonable.

Based on model operation and household surveys, the performance appraisal score for rural infrastructure construction in Sichuan Province was found to be 4.7606. From the perspective of farmers, the performance of rural infrastructure construction is not very good and is inconsistent with China’s increasing infrastructure investments. This result is consistent with Arjuna and Manoj, who found that a lack of infrastructure affects rural residents’ satisfaction because of physical activity in the rural areas of Sri Lanka [41]. However, the longitudinal comparison score for farmer satisfaction was 6.1909, which was the highest among all factors investigated. This case study result represents a typical contribution to the method for estimating the social sustainability of infrastructure [42]. This indicates that the overall rural infrastructure is better than ever, which is consistent with China’s infrastructure investment. In addition, farmers’ willing to invest in rural infrastructure construction was strong with a score of 6.0413, indicating their eagerness to improve rural infrastructure. This is because infrastructure development has a positive influence on agricultural land and regional sustainable development [43]. China’s rural construction over the past 10 years has caused farmers to gain confidence in the construction of rural infrastructure. However, many farmers do not believe existing rural infrastructures are put to full use. The score for impression of full use was 3.3817, which was the lowest among all factors. This suggests that some existing infrastructures are unnecessary, and some are negatively viewed as “vanity projects.” While investment in rural infrastructure has increased, it is not effectively used where it is most needed. In the future, we should pay more attention to the real needs of rural areas and farmers in the construction of rural infrastructure. Regarding environmental perceptions, farmers generally believed that construction did not improve the rural environment (score: 3.9421). It is clear that damage will occur if China does not pay sufficient attention to the environment during the process of rapid infrastructure construction.

The weight of the performance appraisal of rural infrastructure construction reflects the importance of various indexes. This study found that the index weights for overall impression of quality, sense of quality under given costs, complaints to others, complaints to relevant departments, possibility of building information transparency, and possibility of no corruption were the largest at 0.0682. Weight depended on the experience of professionals, and their degree of specialization was high. From their perspective, farmers’ complaints greatly influence the performance appraisal of rural infrastructure construction. Different types of complaints may come from different lifestyles, and they are important in the performance appraisal of rural infrastructure construction [44]. The possibility of building information transparency and of no corruption also greatly influence performance appraisal. We should, therefore, focus on information transparency and on preventing corruption in future rural infrastructure construction. This also highlights the need for better supervision. Experts emphasized the quality of rural infrastructure construction, so the overall impression of quality and the sense of quality under given costs had the highest weight.

This performance appraisal of rural infrastructure construction fully considered farmers’ perceptions and professional advice. At 0.3293, the possibility of building information transparency had one of the highest scores; it was very large for both farmers and experts. This is in line with the conclusion that information transparency has an important influence on the performance appraisal of infrastructure [45]. We can conclude that construction administrative departments should increase the transparency of information regarding rural infrastructure construction in the future. Governments should open all information channels to help villagers understand rural infrastructure construction, and for balance, governments should accept feedback from villagers. The longitudinal comparison score of 0.3285 reflects the fact that rural infrastructure is under constant development. Studying the index of the longitudinal comparison of rural infrastructure from the perspective of experts is very important for performance appraisal and emphasizes the importance of development. The experiences of experts are also very important for urban infrastructure construction [46].

The performance appraisal indexes were selected from the relevant literature as well as discussions with rural students and experts in Sichuan Province. In addition, the relationships between the indexes were judged by 91 experts in Sichuan Province. As such, the conclusions have strong geographical applicability, and different areas may yield different findings.

The performance appraisal of rural infrastructure should also consider aspects such as technology, planning, location, and project management. Since this study evaluated rural infrastructure based only on farmer satisfaction, it has strong subjectivity. In future work, other aspects should be added for a more comprehensive appraisal.

## Conclusion

Based on previous studies, this research established a comprehensive ACSI-ISM-ANP framework for rural infrastructure performance evaluation [37,39,42,46]. A systematic integration of the experiences of infrastructure construction experts and the judgments of infrastructure users was used to evaluate rural infrastructure performance. Overcoming the problem of one-sided performance evaluation in the past, this approach not only satisfies the scientific aspect of performance evaluation but also considers the needs of infrastructure end users.

The performance appraisal score for rural infrastructure in Sichuan Province was determined to be 4.7606. Based on farmer satisfaction, this score is relatively low. According to the data analysis, farmers are most satisfied with the longitudinal comparative perception of rural infrastructure construction. The possibility of participation in rural infrastructure investment is also relatively high. However, farmers have a very bad impression of the function and application of rural infrastructure. They are also dissatisfied with the effect on the environment. From the index of evaluation weights, this study concludes that the performance of rural infrastructure construction affects farmers’ complaints, the possibility of transparency, and the possibility of no corruption a great deal. Moreover, transparency in building information has the highest value, followed by longitudinal comparison.

Appraising the performance of rural infrastructure construction should cover a wide range of aspects. This study conducted the performance appraisal of rural infrastructure construction based only on farmer satisfaction. Thus, the authors intend to gradually consider and study other aspects.

## Supporting information

S1 File (ZIP)
